# SOCIAL INTERACTION AND PSYCHOLOGICAL WELL-BEING OF PERSONS LIVING WITH DEMENTIA IN LONG-TERM CARE

**DOI:** 10.1093/geroni/igac059.2578

**Published:** 2022-12-20

**Authors:** Kyung Hee Lee, Ji Yeon Lee

**Affiliations:** Yonsei University, Seoul, Seoul-t'ukpyolsi, Republic of Korea; Yonsei University, Seoul, Seoul-t'ukpyolsi, Republic of Korea

## Abstract

Although social interaction might play a critical role to improve psychological well-being of old adults with dementia in long-term care, lack of social interaction between staff and residents has been reported. The purpose of this study was to examine whether social interaction is associated with psychological well-being of persons living with dementia during care in long-term care. We analyzed 258 videos from 30 participants with dementia. Each participant was taken nine videos at 0, 3, and 6 months at three care events (i.e., personal care, mealtime, and activity). Social interaction was assessed by quality (i.e., positive, neutral, and negative), type (i.e., verbal, nonverbal, and both), and presence of interaction (i.e., no and yes). Psychological well-being was measured by positive and negative emotional expressions. A mixed model was chosen for the data analysis since these repeatedly measured observation data were nested within subjects. Mixed models showed that positive and neutral interactions were significantly associated with positive emotional expressions of residents after controlling covariates while negative interaction was significantly associated with negative emotional expressions. There was no significance between interaction type and emotional expressions. This study highlights the importance of positive care staff interactions in dementia care. In addition, the institutional efforts to create an environment to reduce negative interactions can be of great help in promoting the psychological well-being of persons living with dementia.

